# Antibacterial properties of chicken intestinal phospholipase A2

**DOI:** 10.1186/1476-511X-10-4

**Published:** 2011-01-12

**Authors:** Aida Karray, Yassine Ben Ali, Youssef Gargouri, Sofiane Bezzine

**Affiliations:** 1 Laboratoire de Biochimie et de Génie Enzymatique des Lipases, ENIS Route de Soukra, 3038 Sfax. University of Sfax -Tunisia

## Abstract

**Background:**

The presence of chicken group-IIA PLA2 (ChPLA2-IIA) in the intestinal secretion suggests that this enzyme plays an important role in systemic bactericidal defence. We have analyzed the bactericidal activity of purified ChPLA2-IIA, on several gram-positive and gram-negative bacteria by using the diffusion well and dilution methods.

**Results:**

ChPLA2-IIA displays potent bactericidal activity against gram-positive bacteria but lacks bactericidal activity against gram negative ones. We have also demonstrated a synergic action of ChPLA2-IIA with lysozyme when added to the bacteria culture prior to ChPLA2-IIA. The bactericidal efficiency of ChPLA2-IIA was shown to be dependent upon the presence of calcium ions and then a correlation could be made to its hydrolytic activity of membrane phospholipids. Interestingly ChPLA2-IIA displays a higher dependence to Ca2+ ions than to Mg2+ions.

**Conclusion:**

We conclude that the main physiological role of ChPLA2-IIA could be the defence of the intestine against bacterial invasions.

## Background

Phospholipase A_2 _(PLA2) comprises a diverse family of enzymes that catalyzes the hydrolysis of glycerophospholipids at the *sn-2 *position to produce free fatty acids and lysophospholipids [1,2]. At present, mammalian secreted PLA2s (sPLA2) are classified into groups I, II, V, and X. Group II sPLA2 has been further classified into five subtypes (Type IIA, IIC, IID, IIE, and IIF) on the basis of their primary sequences. Group IIA PLA2 is one of the key enzymes in the process of inflammation that regulates the synthesis of arachidonic acid and lysophospholipids [3-5]. The structure of human group IIA PLA2 is unusual because of the highly cationic nature of the protein with a great number of positively charged residues (arginine and lysine) spread all over its surface [6]. This provides a molecular explanation for its well established physiological antibacterial activity. Furthermore, tissue and cellular localisations of the enzyme are consistent with its antibacterial role. Indeed, high concentrations of group IIA PLA2 are expressed in Paneth cells of the small intestine [7-11], as well as in human lacrimal cells (and found in tears) [12,13] and in human prostate cells (and found in seminal plasma) [14]. These various localisations are related to possible sites of bacterial invasion into externally-exposed body cavities. The bactericidal potency of rabbit [15], mouse [8], and human [16] group IIA PLA2 is mostly directed against Gram-positive bacteria. However, Harwig and coworkers showed that murine intestinal group IIA PLA2 is bactericidal against some Gram-negative bacteria, such as *Escherichia coli*, *Salmonella, and pseudomonas *[8].

Enzymatic activity (phospholipolysis) is required for the bactericidal activity of mammalian group IIA sPLA2 [8,15,17]. It has been suggested that bacterial envelope sites engaged in cell growth may represent preferential sites for the action of group IIA sPLA2 against Gram-positive bacteria [18].

Overall, bacterial cell wall components, outside of the membrane phospholipids, seem to provide a physical barrier for the access of sPLA2 to their substrates. Furthermore, the cell wall of gram positive bacteria bears a highly anionic charge due to the presence of phosphate diester units of lipotechoic acid. The structure of the gram negative bacteria cell wall is much more complex since it contains 10% to 20% of lipids with a thin layer of peptidoglycan surrounded by an external membrane of phospholipids containing lipopolysaccharides (LPS) and proteins. The bactericidal action of group IIA sPLA2 against Gram-negative bacteria is more difficult to explain than its action against Gram-positive bacteria. In the later case, the sPLA2 pass through the highly anionic cell wall of gram positive bacteria to reach their target phospholipids. The number and the location of positive charges on the surface of the enzyme could be important for the bactericidal activity of sPLA2. The purpose of the present study was to evaluate the possible mechanisms of chicken group IIA PLA2 when killing various antibiotic- resistant or sensitive bacterial strains. For this purpose we used native group IIA PLA2, previously purified from chicken mucosa and we measured its bactericidal properties. Comparative studies were performed using the PLA2 group IB purified from chicken pancreas. We showed that ChPLA2-IIA was more effective than ChPLA2-IB in killing the Gram-positive bacterial. The role of lysozyme as a defensive enzyme has been well documented in vertebrates [19,20] and insects [21-25]. In order to establish if there is a synergistic action between group IIA PLA2 and lysozyme, we tested the antibacterial effect of ChPLA2-IIA against bacteria after pre-incubation with 2 mg/ml (final concentration) of lysozyme."

## Results

### Antibacterial activity of ChPLA2-IIA

We performed the well diffusion methods .to test the antibacterial activity of 13 μg of Ch PLA2-IIA added to 10^8 ^cells of growing culture of gram-positive bacteria: *Bacillus cereus (BC), micrococcus luteus (ML), Brevibacterium flavum(BF), Staphylococcus aureus (SA)*, *Staphylococcus epidermis(SEp), Bacillus subtilis (BS)*, *Enterococcus faecalis (EF)*, and *enterococcus faecium (EFa)*, and gram negative bacteria: *Pseudomonas Aeruginosa*, (*Ps*), *Salmonella *(*S*), and *Klebsielle pneumoniae *(*KP*), *Enterobacter cloacae *(*EC*) and *E. coli (ECo)*. Bacteria were incubated for 24 hours. ChPLA2-IIA was active against closely related Bacillus species, but also some other Gram-positive bacteria (Table 1). Indeed, the enzyme exhibited an important bactericidal effect against *BC*, *BS *and *ML *with a diameter of inhibition higher than 20 mm. A moderate bactericidal effect was obtained with a diameter of inhibition between 15 and 20 mm against *EF *and *BF*. This antibacterial PLA2 activity was much less with an inhibition diameter lower than 15 mm against *EF*, *SA and *SEp. Whereas, ChPLA2-IIA shows no antibacterial activity against Gram-negative bacteria (*EC*, *Ps*, *S*, *KP *and *E Co*).

**Table 1:** Inhibitory spectrum of ChPLA2-IIA on several Gram-positive and gram-negative bacteria.

Bacteria stain	Gram	Sensitivity	IC50 (μg/ml)
*Bacillus cereus*	+	+++	12

*Bacillus subtilis*	+	+++	9.5

*Micrococcus luteus*	+	+++	9

*Brevibacterium flavum*	+	++	14

*Enterococcus faecium*	+	++	-

*Enterococcus faecalis*	+	+	34

*Staphylococcus aureus*	+	+	19

*Staphilococcus epidermidis*	+	+	14

*Enterobacter cloacae*	-	-	-

*Klebsielle pneumoniae*	-	-	-

*Escherchia coli*	-	-	-

*Salmonella*	-	-	-

*Pseudomonas aeruginosa*	-	-	-

The bactericidal level was estimated by measuring the size of inhibition zone of the indicator strain. Inhibitory concentration reducing 50% the bacterial growth of gram(+) bacteria.

Insensitivity (-), low sensitivity (+: Diameter of inhibition < 15 mm), high sensitivity (++: Diameter of inhibition between 15 et 20 mm) and very high sensitivity (+++: Diameter of inhibition > 20 mm).

**Figure 1:**
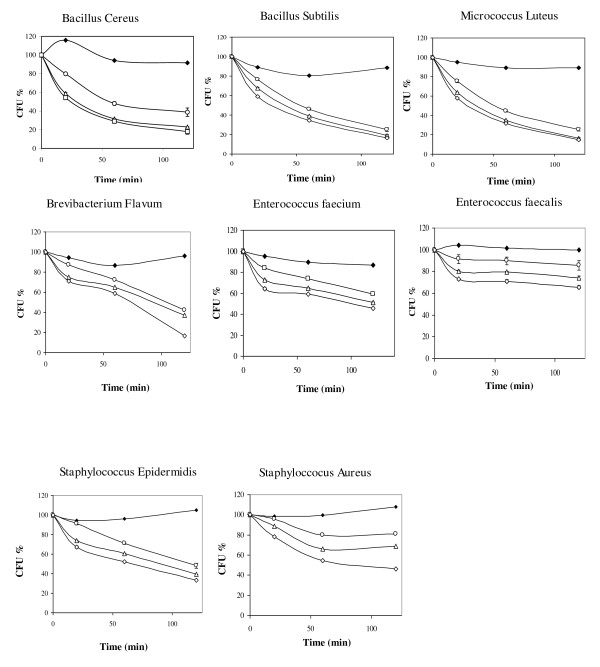
***In vitro *bactericidal activity of chicken sPLA2-IIA**. Samples of gram positive bacterial suspensions were taken after incubation with chPLA2-IIA at various concentrations for 20, 60 and 120 min and thereafter cultured on agar. Bacterial viability was assessed by measuring colony forming ability as described in "Materials and Methods". The results shown in the figure are means of two independent tests. The sPLA2 final concentrations were follows: white circle = 15 μg/ml sPLA2-IIA, white triangle = 30 μg/ml sPLA2-IIA, white square = 45 μg/ml sPLA2-IIA, black square = 50 μg/ml sPLA2-IB.

**Figure 2:**
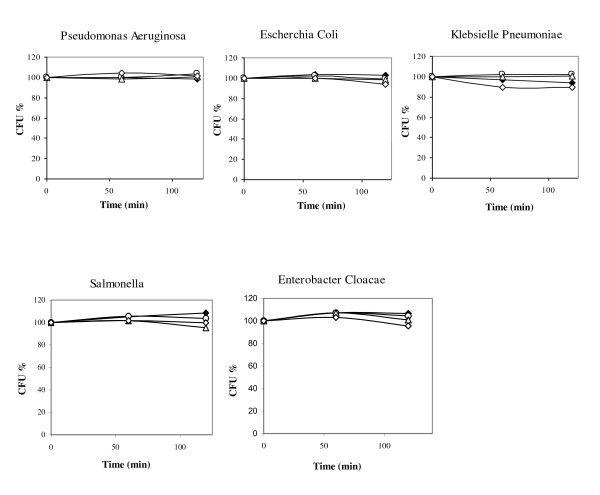
***In vitro *bactericidal activity of chicken sPLA2-IIA**. Samples of gram negative bacterial suspensions were taken after incubation with sPLA2 at various concentrations for 20, 60 and 120 min and thereafter cultured on agar. Bacterial viability was assessed by measuring colony forming ability as described in "experimental procedures". The results shown in the figure are means of two independent tests. The sPLA2 final concentrations were follows: white circle = 15 μg/ml sPLA2-IIA, white triangle = 30 μg/ml sPLA2-IIA, white square = 45 μg/ml sPLA2-IIA, black square = 50 μg/ml sPLA2-IB.

The bactericidal effects of ChPLA2-IIA were also tested by measuring the number of CFU after incubating live bacteria with various concentrations of sPLA2 for various times (see material and methods). As we can see in Figure 1, the decrease of cell viability began slow at the first 10 minutes of incubation. Very effective Gram-positive bactericidal activity was observed for ChPLA2-IIA, even at the lowest concentration tested of 15 μg/ml. The enzyme killed 75% of *BS *and *ML*, 60% of *BC *and 50% of *SEp *after 15 min of incubation. However, 30 μg/ml of ChPLA2-IIA displayed bactericidal activity and killed 90% of *ML *and 80% of *BS *and *BC *after 2 h of incubation. At a final concentration of 45 μg/ml, ChPLA2-IIA killed 85% *BS*, *ML*, *BF*, *BC *and *SEp*, and 50% of *EF *in 2 hours. However, no bactericidal activity was measured against Ps, S, KP, E Co, ML and EC, even at the highest concentration tested (Figure 2).

Pancreatic ChPLA2-IB displayed poor bactericidal activity only against *BF *and *ML *at the highest final concentration tested of 100 μg/ml. In fact, 100 μg/ml (final concentration) ChPLA2-IB killed only 20% of these tow above mentioned bacteria after 1 hour, but was inactive against the other bacteria strains tested.

### Effects of divalent cations

We have tested the effect of Ca2+ ion on the antibacterial activity of ChPLA2-IIA against *ML *and *BS *as described in materiel and methods.

**Figure 3:**
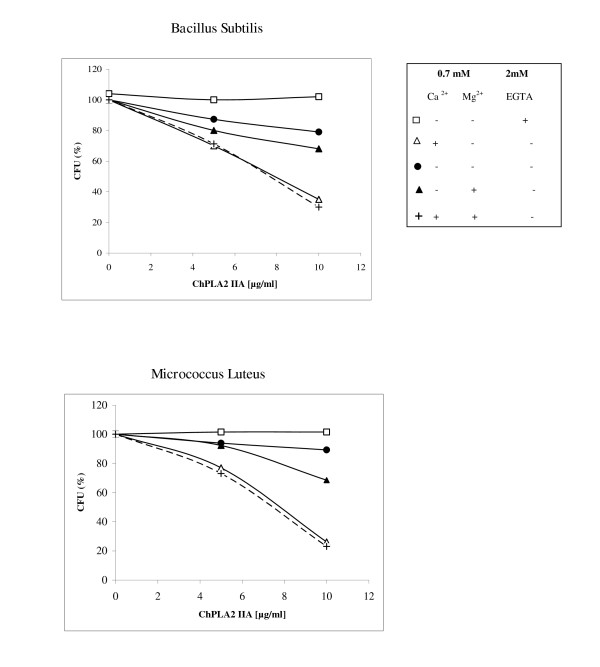
**Calcium dependency of the antibacterial activity**. ChPLA2-IIA was tested against ML and BS bacteria. Strains were incubated with 0.5 and 10 μg/ml (final concentrations) of sPLA2-IIA without or with 0.7 mM divalent cations as indicated. Each symbol represents a mean value from three separate experiments. Results are given as mean values of duplicate determination of CFU.

Figure 3 shows that the addition of 0.7 mM CaCl2 to the enzyme incubated with 10^7 ^bacteria enhanced the bactericidal potency of ChPLA2-IIA between 35% to 79% compared to the bactericidal activity without addition of Ca2+. Whereas addition of 2 mM EGTA, abolished its antimicrobial activity even when the incubation mixture contained 0.7 mM calcium.

"The low activity of ChPLA2-IIA, without purposely added calcium (Figure3, solid circles), is probably due to the presence of calcium in the BHI medium. Interestingly, the presence of 0.7 mM Mg2+ increases the bactericidal effect of ChPLA2-IIA but to a lower extent as compared to that of Ca2+ ions. However both Ca2+ ions and Mg2+ ions added to the incubation mixture, gave the same ChPLA2-IIA activity compared to the pre-incubation mixture with the Ca2+ ions only. This observation suggests that ChPLA2-IIA possesses a higher affinity to Ca2+ ions than that to Mg2+ ions. Similar results were obtained with human and murine group IIA sPLA2 [12]. Thus antibacterial activity of ChPLA2-IIA seems to be related to its enzymatic activity.

### Synergy between ChPLA2-IIA and lysozyme activities

**Figure 4:**
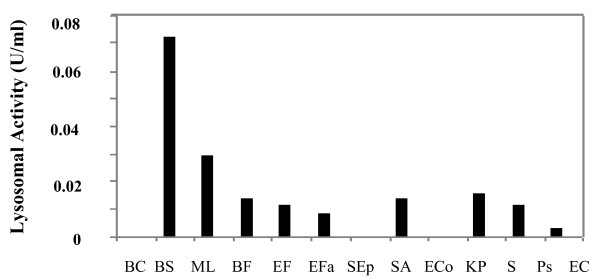
**Initial rates of hydrolysis of lysozyme against several lyophilized bacteria**. Substrate suspensions (10^7 ^cells/ml) were incubated with 2.5 mg/ml of lysozyme. One enzyme unit was defined as the amount causing decrease of 0.1 absorbance units at 540 nm in the reaction for 1 min at 25°C. The essay was performed in triplicate per sample.

Lysozyme hydrolyse the β-1,4 glucosidic linkage between N-acetyl muramic acid and N-acetyl glucosamine of peptidoglycan, a cell wall component of bacteria. We first measured lysozyme bactericidal activity against the full set of gram+ and gram- bacteria. A highly variable ability of lysozyme to hydrolyse suspension of bacteria was observed (Figure 4). The highest Lysozyme activity was much higher against gram positive bacteria especially against suspension of *ML *and *BS*. A fast decrease of cells viability was observed during the first minutes.

**Figure 5:**
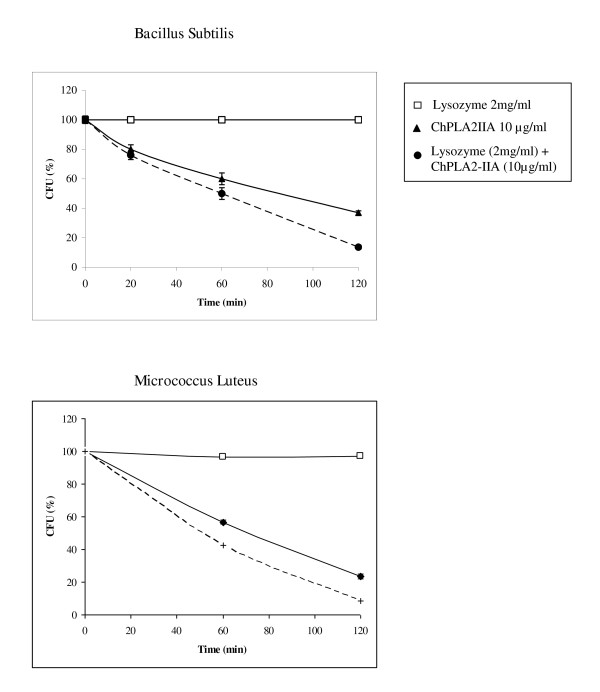
**Effect of lysozyme pre-treatment on the ability of ChPLA2-IIA to kill cell suspensions of BS and ML**. Cell suspensions of *BS *and *ML *(10^7^/ml) were incubated with 2.5 mg/ml of lysozyme for 2 min prior to then addition of ChPLA2-IIA (45 μg/ml). Samples were taken at 20, 60 and 120 min and plated on BHI agar and grown for 18 h.

To evaluate possible synergic effects between lysozyme and ChPLA2-IIA bactericidal activities, we have chosen ML and BS for their high sensitivity to lysozyme. After two minutes of pre-treatment with lysozyme, the addition of 15 μg/ml (final concentration) of sPLA2-IIA increases the ability of the PLA2 to kill the bacteria (Figure 5). The IC50 values of ChPLA2-IIA decrease from 9 μg/ml to 4.5 μg/ml against ML and from 9.5 μg/ml to 6 μg/ml against BS. The inhibition ratio of *ML *increases from 85% to reach 91% after 2 h. This ratio is defined as the rate of the live bacteria number without addition of the PLA2 devised by the live bacteria number counted after the PLA2 addition.

## Discussion

### Bactericidal activity of chicken sPLA2

Chicken intestinal sPLA2 is an 18 kDa molecule composed of 134 amino acid residues. It has been purified from the intestine mucosa (Karray A, Frikha F, Ben Ali Y, Gargouri Y, Bezzine S: Purification and Biochemical Characterization of Cationic Chicken Intestinal phospholipase A2, submitted). In this previous work, we demonstrated that ChPLA2-IIA hydrolyzes phosphatidylglycerol (which is the main phospholipid present in microbial membranes) more rapidly than phosphatidylcholine (abundant in mammalian cell membranes)[26]. In the present work, we have demonstrated that only 15 μg/ml (final concentration) of ChPLA2-IIA is able to kill 75% of *BS *and *ML*, and IC50 values are 9 μg/ml and 9.5 μg/ml, respectively. The same amount of enzyme kills 60% of *BC *with an IC50 value of 12 μg/ml. ChPLA2-IIA was less active against *BF*, *SA*, *SEp *and *EF *with IC50 values of 12,14,19 and 34 μg/ml, respectively. Whereas, even at a final concentration of 50 μg/ml, ChPLA2-IIA is inactive against gram negative bacteria.

The cell wall of gram positive bacteria contains a dense peptidoglycan array, which bears a high anionic charge due to the presence of phosphate diester units of lipoteichoic acid. The sPLA2 must go through the highly anionic cell wall of Gram positive bacteria to reach the phospholipids membrane target. ChPLA2-IIA, with an electrostatic potential of +16 (+23; -7) based on the total number of Lys, Arg, His, Asp and Glu, can bind highly anionic bacterial cell walls [27], in contrast to the PLA2 group IB with a net tabulated charge of +1 (+19, -18).

A previous study showed the ability of human sPLA2-IIA to kill *Bacillus antracis*, the etiological agent of antrax [28]. The authors showed that both germinated *B. anthracis *spores and encapsulated bacilli were sensitive to the bactericidal activity of human sPLA2-IIA *in vitro*. Moreover, the human PLA2-IIA appears to be a major antibacterial factor against Gram positive bacteria in human acute phase serum. The bactericidal activity of human PLA2-IIA was also shown against *Staphylococcus aureus *and *Listeria monocytogenes *in serum samples collected from patients with acute bacterial infections and healthy control subjects [29].

Pancreatic ChPLA2-IB, also tested, at a high final concentration of 50 μg/ml didn't show any bactericidal activity, similar to the porcine, human, and mouse pancreatic sPLA2 [27,30]. Infact, 0.5 μg/ml (final concentration) of human PLA2-IIA killed over 90% of gram positive bacteria, but was inactive against *E.coli *even at higher concentration. However, human and mouse group-IB PLA2 displayed modest bactericidal activity against *Listeria *monocytogene [30].

Enzymatic activity is required for the bactericidal activity of mammalian group IIA sPLA2 against Gram-positive [8,13] and Gram-negative [18] bacteria. It has been suggested that bacterial envelope sites engaged in cell growth may represent preferential sites for the action of group IIA sPLA2 against Gram-positive bacteria [15]. Moreover, bacteria are more resistant to group IIA sPLA2 in the stationary phase than in the logarithmic growth phase, suggesting that these microorganisms are more susceptible to sPLA2 when they are dividing [15]. Overall, bacterial cell wall components outside of the phospholipid membrane seem to provide a barrier for the access of sPLA2 to the phospholipid membrane surface. However, the bactericidal action of sPLA2 group IIA against Gram negative bacteria is more complex than its action against Gram-positive ones.

### Calcium dependency of ChPLA2 activity

We showed that bactericidal activity of ChPLA2-IIA is calcium dependant since it was totally inhibited by EGTA. This result suggests that the enzymatic activity of ChPLA2-IIA was critical for its bactericidal properties. Qu and Lehrer [12] showed in previous work that 2 mM EGTA abolished the bactericidal effect of human tears and greatly reduced their bactericidal activity against *Streptococci *and *Listeria monocytogene*. This confirms that the antibacterial activity of PLA2 group IIA from different species is linked to their enzymatic hydrolysis of membrane phospholipids. Interestingly, ChPLA2-IIA shows a much higher affinity to Ca2+ ions as compared to Mg2+ ions since we found the same bactericidal activity with Ca2+ ions alone or with Ca2+ and Mg2+ ions together."

### Synergic effect of ChPLA2-IIA with lysozyme

Our data provide compelling evidence that ChPLA2-IIA is mostly responsible for killing a broad spectrum of gram-positive bacteria not withstanding the presence of lysozyme at higher concentrations. This inference is supported by several lines of evidence. Firstly, much lower concentrations of purified ChPLA2-IIA (15 μg/ml final concentration) than those of lysozyme (2.5 mg/ml final concentration) showed potent bactericidal activity against the gram positive bacteria tested. Secondly, lysozyme acts in synergy with the ChPLA2-IIA since a prior treatment of the bacteria with 2 mg/ml (final concentration) of lysozyme increases the ability of the ChPLA2-IIA to hydrolyse suspensions of intact ML. Thus, the treatment of ML with lysozyme probably disrupts the cell wall to allow a better access of the ChPLA2-IIA to the cell membrane in order to hydrolyse the phospholipids.

### Sequence analysis of ChPLA2-IIA

In the present work the IC50 of ChPLA2-IIA was ranging between 9 μg/ml (final concentration) and 35 μg/ml (final concentration). However, IC50 of human and mouse PLA2-IIA were ranging between 1 ng/ml to 0.5 μg/ml [12,30]. These data indicate that ChPLA2-IIA is less potent at killing Gram positive bacteria than human PLA2-IIA. In a previous work, Koduri et al [30] showed that the triple site mutations R7E/K10E/K16E and K74E/K87E/R92E of basic residues on the putative membrane binding surface of human PLA2-IIA decreased greatly the antibacterial activity of the enzyme. These basic residues reinforce the binding of the human PLA2-IIA to the highly anionic phosphatidyl glycerol which is abundant in bacterial membranes. Aminoacid sequence alignment of human and chicken PLA2-IIA shows that R7 and R92 are replaced by I and Q, respectively which may explain the lower potency of chicken PLA2-IIA at killing gram positive bacteria.

Interestingly, *Staphylococcus aureus *responds to PLA2 attack by continued phospholipid synthesis, and thus the fate of the bacterium exposed to PLA2 depends on the relative rates of phospholipid degradation and synthesis [31].

## Conclusion

ChPLA2-IIA purified from the intestine mucosa possesses an antibacterial activity against all gram + bacteria tested and especially against ML, BS and BC but it was much less active against gram - bacteria. On the one hand, the antibacterial property of ChPLA2-IIA is probably closely related to its enzymatic activity since CaCl2 and MgCl2 (0.7 mM) are required and these activities are not observed in the presence of an ion chelator (2 mM EGTA). Moreover, ChPLA2-IIA possesses a much higher affinity to Ca2+ ions than to Mg2+ ions. Furthermore, ChPLA2-IIA acts in synergy with lysozyme. A prior treatment of bacteria with 2 mg/ml (final concentration) lysozyme disrupts the cell wall to allow a better access of the ChPLA2-IIA to the cell membrane.

## Materials and methods

### Materials

Bovine serum albumine (BSA), sodium taurodeoxycholate (NaTDC), were from Sigma Chemical (St. Louis, USA). Ethylene Diamine Tetra Acetic acid (EDTA) was from Sigma-Aldrich (St. Quentin-Fallavier, France). Brain Heart Infusion (BHI) was from Hispanlab, S.A (Madrid).

### Enzyme samples

ChPLA2-IIA was purified from chicken intestine mucosa (Karray A, Frikha F, Ben Ali Y, Gargouri Y, Bezzine S: Purification and Biochemical Characterization of Cationic Chicken Intestinal phospholipase A2, submitted). The specific enzymatic activity of ChPLA2-IIA using a pH state assay is about 160 U/mg measured at optimal conditions (pH 9.0 and 40°C) in the presence of 10 mM NaTDC and 10 mM CaCl_2 _using egg phosphatidylcholine as substrate. The chicken pancreatic sPLA2, taken as a negative control was also purified in the laboratory [32]. Lysozyme from chicken egg white was purchased from sigma (France).

### Bactericidal Assays

#### Diffusion well method

The antibacterial activity of ChPLA2-IIA was checked by well diffusion method [33,34]. Briefly, bacteria were cultivated in BHI medium at 37°C for 3 h. A basal layer of BHI containing 2-5% agar, was poured in Petri dishes. When plates were dried, 5 ml of soft BHI (0-7% agar) containing 10^7 ^cells of the indicator strain were overlaid.

Then, wells were punched in the agar plate and filled with 5 μg of test samples. After 18 h of incubation at 37°C, the diameter of the zone of inhibition was measured. One arbitrary unit (AU) of antibacterial activity was defined as the amount of ChPLA2-IIA sufficient to give a zone of inhibition around the well.

The bacteria used were staphylococcus. *aureus (SA)*, staphylococcus epidermidis (*SEp*), *Bacillus cereus *(*BC*), *Bacillus subtilis *(*BS*), Micrococcus Luteus (*ML*), *Enterococcus faecalis *(*EF*), *Enterococcus faecium *(*EFa*), *Enterobacter cloacae *(*EC*), *Brevibacterium flavum *(*BF*), *Pseudomonas Aeruginosa*, (*Ps*), *Salmonella *(*S*), *Klebsielle pneumoniae *(*KP*) and *E. coli (ECo) *(Table 1).

#### Dilution method

Bacterial viability was assessed by measuring colony-forming ability of bacteria incubated in the absence or presence of PLA2 for various times. Bacteria were first incubated in 100 ml of BHI medium at 37°C for 3 h30 min. Thereafter, they were centrifuged at 200 rpm for 10 min at 4°C *in *an Eppendorf tube. Cell pellets were suspended in 10 ml of sterile saline buffer and centrifuged as previously. This step was repeated twice. Then, cells were suspended in saline buffer, and the OD_650 _nm was adjusted to 0.5 (10^8 ^bacteria/ml). OD_650 _was measured with an Ultrospec III spectrophotometer (Pharmacia, Piscataway, NJ). Tow microliter of the suspension was added to 500 μl of Tris buffer (50 mmol/L Tris, and 10 mmol/L Ca2+, 10 mg/ml bovine serum albumin, pH 7.4) and shaken. 20 μl of the bacterial suspension bacteria in tris buffer (20 mmol/liter Tris, 10 mmol/liter Ca2+, 10 mg/ml bovine serum albumin, pH 7.4) was added to 20 μl of sPLA2 solution. A mixture of 20 μl of bacterial suspension and 20 μl of sterile saline served as a sPLA2 negative control. The resulting solutions thus contained 45, 30, 15, and 0 μg/ml sPLA2. The sPLA2 negative control for ChPLA2 contained the same amount of buffer as the 45 μg/ml sPLA2 solutions. The solutions were incubated with bacteria at 37°C. Samples were taken at 20, 60, and 120 min and plated on brain heart infusion agar and grown for 18 h to measure colony-forming units (CFU). The bactericidal tests were performed twice on each bacterium and enzyme, and the results are given as mean values of duplicate determinations.

#### Effects of divalent cations on the ChPLA2-IIA antibacterial activity

To determine the degree of which ChPLA2-IIA was calcium and/or magnesium dependent, we evaluated the effect of addition of 0.7 mM CaCl2, or MgCl2 on the bactericidal activity. The addition of 2 mM EGTA, a selective divalent cations chelator was also performed. Incubation mixtures contained 2 10^7 ^bacteria/ml were washed twice with sterile salt solution and supplemented with 0.7 mM CaCl2, and (or) 0.7 mM MgCl2, 10 mg/ml sterile bovine serum albumin, and the appropriate amount of ChPLA2-IIA. Reactions were carried out at 37°C for up to 2 h. At various time points, aliquots were taken and serially diluted into sterile saline buffer and plated in agar dishes to determine CFU. An aliquot was supplemented with 2 mM EGTA.

#### Lysozyme activity assay

The lysozyme activity was assayed based on the method of Thammasirirak et al. [35], using lyophilized cells of all stains used previously as a substrate. Cell suspension in 0.1 M sodium phosphate buffer, pH 7.0, was diluted and adjusted to OD_540 _of 0.7-0.8 at 540 nm. Enzyme solution (100 μL) was added to 3 mL of cell suspension. The enzymatic activity was evaluated from the decrease of absorbance at 540 nm for 3 min. One enzyme unit was defined as the amount of lysozyme causing a decrease of 0.1 absorbance units in the reaction mixture for 1 min at 25°C. The assay was performed in triplicate per sample.

#### Effect of lysozyme pretreatment on the ability of ChPLA2-IIA to hydrolyse cell suspensions

Cell suspensions (10^7 ^cells/ml) of several bacteria, were incubated with 2.5 mg/ml of lysozyme for 2 min prior to the addition of 45 μg/ml of the ChPLA2-IB and ChPLA2-IIA. Samples were incubated for 20, 60 and 120 min and then plated for 24 h to measure colony-forming units (CFU).

## Abbreviations

ChPLA2-IIA: chicken group IIA PLA2; ChPLA2-IB: chicken group IB PLA2; LPS: lipopolysaccharid; EDTA: Ethylene Diamine Tetra Acetic acid; CFU: colony-forming units; *SA*: Staphylococcus *aureus*; *SEp*: Staphylococcus epidermidis; *BC*: *Bacillus cereus*; *BS*: *Bacillus subtilis*; *ML*: Micrococcus Luteus; *EF*: *Enterococcus faecalis*; *EFa*: *Enterococcus faecium*; *EC*: *Enterobacter cloacae*; *BF*: *Brevibacterium flavum*; *Ps*: *Pseudomonas Aeruginosa*; *S*: *Salmonella*; *KP*: *Klebsielle pneumoniae *and *ECo*: *E. Coli*.

## Competing interests

The authors declare that they have no competing interests.

## Authors' contributions

AK carried out all the studies, analyzed the data and drafted the manuscript. YBA helped with the analysis of the data and to correct the manuscript. YG helped with the discussion of the data and the correction of the manuscript. SB participated in the study design and helped to draft the manuscript. All authors have read and approved the final manuscript.
